# Stromal SPOCK1 supports invasive pancreatic cancer growth

**DOI:** 10.1002/1878-0261.12073

**Published:** 2017-06-05

**Authors:** Veronique L. Veenstra, Helene Damhofer, Cynthia Waasdorp, Anne Steins, Hemant M. Kocher, Jan P. Medema, Hanneke W. van Laarhoven, Maarten F Bijlsma

**Affiliations:** ^1^ Laboratory for Experimental Oncology and Radiobiology Center for Experimental and Molecular Medicine Academic Medical Center and Cancer Center Amsterdam The Netherlands; ^2^ Centre for Tumour Biology Barts Cancer Institute Queen Mary University of London UK; ^3^ Department of Medical Oncology Academic Medical Center University of Amsterdam the Netherlands; ^4^ Present address: Biotech Research & Innovation Centre Copenhagen Denmark

**Keywords:** extracellular matrix, pancreatic ductal adenocarcinoma, SPOCK1, stroma, transforming growth factor‐beta, tumor cell invasion

## Abstract

Pancreatic ductal adenocarcinoma (PDAC) is marked by an abundant stromal deposition. This stroma is suspected to harbor both tumor‐promoting and tumor‐suppressing properties. This is underscored by the disappointing results of stroma targeting in clinical studies. Given the complexity of tumor–stroma interaction in PDAC, there is a need to identify the stromal proteins that are predominantly tumor‐promoting. One possible candidate is SPOCK1 that we previously identified in a screening effort in PDAC. We extensively mined PDAC gene expression datasets, and used species‐specific transcript analysis in mixed‐species models for PDAC to study the patterns and driver mechanisms of SPOCK1 expression in PDAC. Advanced organotypic coculture models with primary patient‐derived tumor cells were used to further characterize the function of this protein. We found SPOCK1 expression to be predominantly stromal. Expression of SPOCK1 was associated with poor disease outcome. Coculture and ligand stimulation experiments revealed that SPOCK1 is expressed in response to tumor cell‐derived transforming growth factor‐beta. Functional assessment in cocultures demonstrated that SPOCK1 strongly affects the composition of the extracellular collagen matrix and by doing so, enables invasive tumor cell growth in PDAC. By defining the expression pattern and functional properties of SPOCK1 in pancreatic cancer, we have identified a stromal mediator of extracellular matrix remodeling that indirectly affects the aggressive behavior of PDAC cells. The recognition that stromal proteins actively contribute to the protumorigenic remodeling of the tumor microenvironment should aid the design of future clinical studies to target specific stromal targets.

**Abbreviations**
EMT: epithelial‐to‐mesenchymal transition
FAP: fibroblast activation protein
IL‐1: interleukin 1
MEF: mouse embryonic fibroblast
PDAC: pancreatic ductal adenocarcinoma
SHH: Sonic Hedgehog
SMAD4: mothers against decapentaplegic homolog 4
SPARC: secreted protein acidic and cysteine rich
*SPOCK1*: human gene/transcript nomenclature
*Spock1*: mouse gene/transcript nomenclature
SPOCK1: Sparc/Osteonectin, Cwcv And Kazal‐Like Domains Proteoglycan
TGF‐β: transforming growth factor‐beta
αSMA: alpha smooth muscle actin

## 1 Introduction

Pancreatic ductal adenocarcinoma (PDAC) is the deadliest form of common cancer (Rahib *et al*., 2014). Factors that contribute to its lethality include an aggressive growth, high intrinsic resistance to (chemo)therapeutics, and diagnosis at stages at which the disease is no longer amenable to curative treatment (Ghaneh *et al*., 2007; Hidalgo, 2010). Another feature that is suspected to contribute to the poor outcome of PDAC is the desmoplastic reaction, an extreme accumulation of nonepithelial cells and material around the tumor cells (Waghray *et al*., 2013). These include cancer‐associated fibroblasts, activated stellate cells, immune cells, but also the deposition of proteins like collagen and fibrinogen that make up the extracellular matrix. Together, these are known as the *stroma*. In most cases of PDAC, the stromal fraction vastly outnumbers the epithelium, and the bulk of the tumor is typically not made up of tumor cells.

Histopathological assessment of activated stroma has been shown to correlate with poor survival, and a wealth of preclinical work has corroborated this notion of a strictly tumor‐promoting role for the stroma (Fujita *et al*., 2010; Hwang *et al*., 2008; Kadaba *et al*., 2013). For instance, the stiff mechanical properties of the stroma reduce the perfusion of PDAC tumors and this negatively affects delivery of chemotherapeutics and also oxygen, causing hypoxia. Furthermore, stromal cells have been described to act as chaperones for tumor cells that disseminate from the primary tumor, presumably providing a niche for malignant cells that would otherwise be vulnerable during transit (Coleman *et al*., 2014). In addition, we and others have previously shown that the stroma provides a wide array of ligands that act *in trans* to support tumor cell growth (Damhofer *et al*., 2013).

Recent clinical trials using stroma‐targeting agents have failed to make good on the promise of preclinical work; none of these trials have been reported to show favorable responses, and one trial was interrupted following accelerated disease progression in the arm receiving the experimental stroma‐targeting agent (BusinessWire, 2014). A tentative explanation for this has come from later experimental work, which demonstrated that the ablation of stroma from PDAC mouse models resulted in enhanced aggressive growth and chemoresistance of the tumor cells (Lee *et al*., 2014; Ozdemir *et al*., 2014; Rhim *et al*., 2014). This is now assumed to also occur in patients. It is possible that the mechanical properties of the stroma keep the tumor cells confined and in place, but it is also likely that stromal trans‐signaling molecules exist that keep tumor cells relatively differentiated and inactive. Which of the stromal contributions are required to keep the tumor relatively indolent is not known. Conversely, which stromal factors have a specifically tumor‐promoting role is also not clear.

We have previously performed a screen for stromal targets of tumor cell‐derived Sonic Hedgehog (SHH), a developmental protein important for the maintenance of stroma in PDAC (Damhofer *et al*., 2013). From this screen, several extracellular genes were identified that were prognostic, and likely to support tumor growth. One such gene was Sparc/osteonectin, Cwcv and Kazal‐like domains proteoglycan (SPOCK1). SPOCK1 is a glycoprotein, highly similar to SPARC, a well‐studied and characterized protein in the context of PDAC tumor growth (Hidalgo *et al*., 2015). Recently, the significance of SPOCK1 for tumor growth, apoptosis, epithelial‐to‐mesenchymal transition (EMT), and metastasis has been reported for tumor types other than PDAC (Fan *et al*., 2016; Li *et al*., 2013; Ma *et al*., 2016; Miao *et al*., 2013; Shu *et al*., 2015; Yang *et al*., 2016; Yu *et al*., 2016). In these tumors, SPOCK1 appears to predominately be expressed in the epithelial fraction. We now demonstrate that in PDAC, the expression of SPOCK1 is stromal rather than epithelial. We show that its expression is driven by tumor cell‐derived transforming growth factor‐beta (TGF‐β) and that the function of SPOCK1 is to translate the reception of this ligand into stromal support for tumor cell growth and migration via the modulation of the extracellular collagen matrix.

## 2 Materials and methods

### 2.1 Expression analysis

Datasets used were as follows (first author and contact name for GEO or ArrayExpress submission are listed): tumor expression data: GSE15471, Badea (Badea *et al*., 2008); GSE16515, Pei/Wang (Pei *et al*., 2009); GSE17891, Collisson/Sadanandam (Collisson *et al*., 2011); GSE21501, Stratford/Yeh (Stratford *et al*., 2010); GSE28735, Zhang/Hussain (Zhang *et al*., 2013); GSE36924, Pérez‐Mancera/Wu (Bailey *et al*., 2016; Perez‐Mancera *et al*., 2012). Cell line expression data: GSE21654, Maupin/Haab (Maupin *et al*., 2010); GSE36133, Barretina/Stranksy (Barretina *et al*., 2012); GSE57083, Wappett; E‐MATB‐783, Garnett/McDermott (Garnett *et al*., 2012). Microdissected tissue expression data: E‐MEXP‐1121, Pilarsky (Pilarsky *et al*., 2008). Gene expression data were collected and processed for use in the AMC in‐house R2: Genomics Analysis and Visualization Platform (http://r2.amc.nl). For visualization of gene expression, data were imported in rstudio (RStudio Inc, Boston, MA, USA) and plotted using ggplot2, or plotted in graphpad prism (Graphpad Software Inc, La Jolla, CA, USA).

### 2.2 Gene set enrichment analysis

gsea software (Broad Institute, Cambridge, MA, USA) was downloaded from the Broad Institute website (http://www.broad.mit.edu/gsea/) and gene sets were obtained from the Molecular Signature Database (MSigDB) and the Kyoto Encyclopedia of Genes and Genomes (KEGG). Expression datasets were assembled with annotated gene names (.txt), samples were dichotomized for median *SPOCK1* expression to yield phenotype label files (.cls), and gene sets were assembled (.gmx). Two thousand permutations were run on the phenotype. Datasets were not collapsed to gene symbols in the gsea software.

### 2.3 Tissue culture

Mouse embryonic fibroblasts (MEFs; kind gift from Matthew Scott, Stanford University) and PANC‐1 cells (ATCC) were cultured in DMEM containing 8% FBS, l‐glutamine (2 mm), penicillin (100 units·mL^−1^), and streptomycin (500 μg·mL^−1^) according to routine cell culture. The primary patient‐derived cell line 67 was cultured in IMDM containing 8% FBS, l‐glutamine (2 mm), penicillin (100 units·mL^−1^), and streptomycin (500 μg·mL^−1^). For cocultures, fibroblasts were seeded in a 1 : 1 ratio with tumor cells at a total amount of 20 000 cells·cm^−2^ and cultured for 7 days. Prior to subsequent analyses, cells were imaged on a Zeiss AxioVert microscope (Jena, Germany).

### 2.4 Lentiviral gene silencing

Lentivirus was produced by transfecting HEK293T cells with Mission TRC library *pLKO* transfer plasmids together with the packaging plasmids *pMD2.G* and *psPAX2* using calcium phosphate. TRC clone numbers used were as follows: 0000079969 and 0000079971. As a control, the *shc002* scrambled plasmid was used. Forty‐eight and 72 h after transfection, supernatant was harvested and 0.45 μm filtered (Millipore, Billerica, MA, USA). 60% confluent MEFs were transduced with lentivirus and 5 μg·mL^−1^ polybrene (Sigma, St. Louis, MO, USA) overnight. Two days after transduction, MEFs were selected with 1 μg·mL^−1^ puromycin (Sigma).

### 2.5 Establishment of primary PDAC cell lines

The collection of patient material was approved by the institute's medical ethical committees (AMC 2014_181), and performed according to the guidelines of the Helsinki Convention. Signed informed consent was always obtained. Grafting of mice with patient material was performed according to the protocols approved by the Animal Experiment Ethical Committee (DTB102348). All surgical procedures were performed under isoflurane anesthesia. For detailed description of primary cell line isolation, propagation, and characterization, see Damhofer *et al*. (2015).

### 2.6 Quantitative reverse transcriptase PCR

Following stimulations or cocultures, cells were harvested with trypsin/EDTA, and RNA was isolated using the NucleoSpin RNA isolation kit (Macherey‐Nagel, Düren, Germany) according to the manufacturer's protocol. cDNA was synthesized using Superscript III reverse transcriptase (ThermoFisher, Waltham, MA, USA). Quantitative PCR was performed using Sybr Green (Roche, Penzberg, Germany) on a Lightcycler LC480 II (Roche). Data were normalized to *GAPDH/Gapdh* transcript levels according to the comparative threshold cycle (Cp) method. Primer sequences used were as follows: *hGAPDH* Fw, aatcccatcaccatcttcca; *hGAPDH* Rv, tggactccacgacgtactca; *hSPOCK1* Fw, aaagcacaaggcagaaagga; *hSPOCK1* Rv, gggtcaagcaggaggtcata; *mGapdh* Fw, ctcatgaccacagtccatgc; *mGapdh* Rv, cacattgggggtaggaacac; *mSpock1* Fw, tgtgtgacccaggactacca; *mSpock1* Rv, tccaagccagtgtttgtgag; *mGli1* Fw, acacgggtgagaagccttac; *mGli1* Rv, ggatctgtgtagcgcttggt; *mPtch1* Fw, gctacgactatgtctctcacatcaact; *mPtch1* Rv, ggcgacactttgatgaacca.

### 2.7 Ligand stimulation experiments

IL‐1β was from Miltenyi; IL‐1α and HGF were from R&D; bFGF and EGF were from TEBU‐BIO; TGF‐β was from Peprotech (Rocky Hill, NJ, USA). ShhN was made by transfecting 293T cells with *ShhN* in pRK5 (from Genentech, South San Francisco, CA, USA) and after transfection, incubating cells in DMEM containing 0.5% FBS. Prior to the addition of ligands, cells were switched to 0.5% FBS culture medium for 16 h. Ligands were added for 24 h.

### 2.8 Lentiviral cell labeling

*pLeGO‐V2* with *Venus* (plasmid #27340, Addgene (Weber *et al*., 2011)) was used for lentivirus production as described under Section 2.4. After overnight transduction, cells were cultured for 72 h before sorting for Venus‐positive cells on a BD FACSAria III.

### 2.9 Flow cytometry

Cells were harvested with trypsin/EDTA and washed in FACS buffer (1% FBS/PBS). Cells were analyzed with 1 μg·mL^−1^ PI and 10 μL CountBright absolute counting beads (ThermoFisher, Waltham, MA, USA), prepared following the manufacturer's instructions, and analyzed on FACSCanto II (BD). Data were analyzed using flowjo v10 (FlowJo LLC, Ashland, OR, USA). From the PI‐negative fraction, the counts in the Venus channel were analyzed and in the PerCP channel bead numbers were counted. The total amount of beads added was divided by the beads acquired to determine the multiplication factor required to accurately determine the total amount of Venus‐positive cells in the culture.

### 2.10 Organotypic cultures

Organotypic cultures were performed according to Kadaba *et al*. (2013). Tumor cells and fibroblasts were plated in a 1 : 2 ratio on top of the gels, solidified on nylon sheets, and grids placed at an air–liquid interphase. Medium was replaced twice weekly. After culturing, gels were processed for both immunofluorescence (IF) and immunohistochemistry (IHC). Gels were fixed with 4% paraformaldehyde for 18 h after 1 week or 3 weeks, incubated in 20% sucrose, and mounted in OCT (Tissue‐Tek) for further processing for IF. For IHC, gels were incubated in 70% ethanol after fixation, and processed according to the standard procedures for paraffin embedding.

### 2.11 Immunohistochemistry and staining

Tissue slides were stained as previously described on the paraffin‐embedded slides (Damhofer *et al*., 2015). Antibodies and dilutions used were CK19 1 : 500 (MU246‐UC; Biogenex), CXCR4 1 : 400 (Ab124824; Abcam, Cambridge, UK), Ki67 1 : 2000 (SAB5500134; Sigma). For picrosirius red staining, slides were deparaffinized, stained in a 0.1% picrosirius red solution (Sigma) in saturated picric acid for 1 h, and washed three times with 0.1 m acetic acid solution. All slides were imaged on an Olympus BX51 (Tokyo, Japan). Quantification of Ki67 staining was performed with Fiji count particles (Schindelin *et al*., 2012), after DAB/H color deconvolution. For IF images, slides were cut at 10 μm, mounted in Prolong Gold (ThermoFisher), and imaged on an EVOS fluorescence microscope (ThermoFisher). For collagen staining in tissue culture vessels, cells were cultured as described and after 7 days of coculture washed with PBS three times prior to fixation in 4% paraformaldehyde for 15 min. Following three washes with PBS, cells were stained for their collagen deposition as described above for 18 h and were equally treated as the tissue slides. Cells were subsequently imaged on an Olympus BX51. Quantifications of the percentage of Ki67‐positive nuclei, width of HE staining, or percentage of Venus‐positive cells were performed using Fiji package of imagej.

## 3 Results

### 3.1 SPOCK1 is upregulated in pancreatic cancer and its expression is confined to the stroma

Using cocultures of human tumor cells and mouse fibroblasts to model the stroma, followed by species‐specific RNA‐Seq analysis, we have previously identified mouse *Spock1* as a stromal target gene of tumor cell‐derived SHH (Damhofer *et al*., 2013). In our previous screen hit selection, we included only those genes that were prognostic in the Badea *et al*.'s (2008) PDAC cohort. To validate that *SPOCK1* expression is also prognostic in other cohorts, we dichotomized patients included in additional expression datasets by median and performed survival analysis (Stratford *et al*., 2010; Zhang *et al*., 2013) (Fig. S1A). In the Stratford *et al*.'s cohort, *SPOCK1* expression higher than median correlated with poor prognosis. Survival analysis on groups dichotomized by scanning for the best prognostic separation yielded highly significant differences in survival outcome in both cohorts (Fig. S1B). These results demonstrate that SPOCK1 is correlated with poor prognosis in multiple datasets and that a stromal gene can be strongly prognostic.

**Figure 1:**
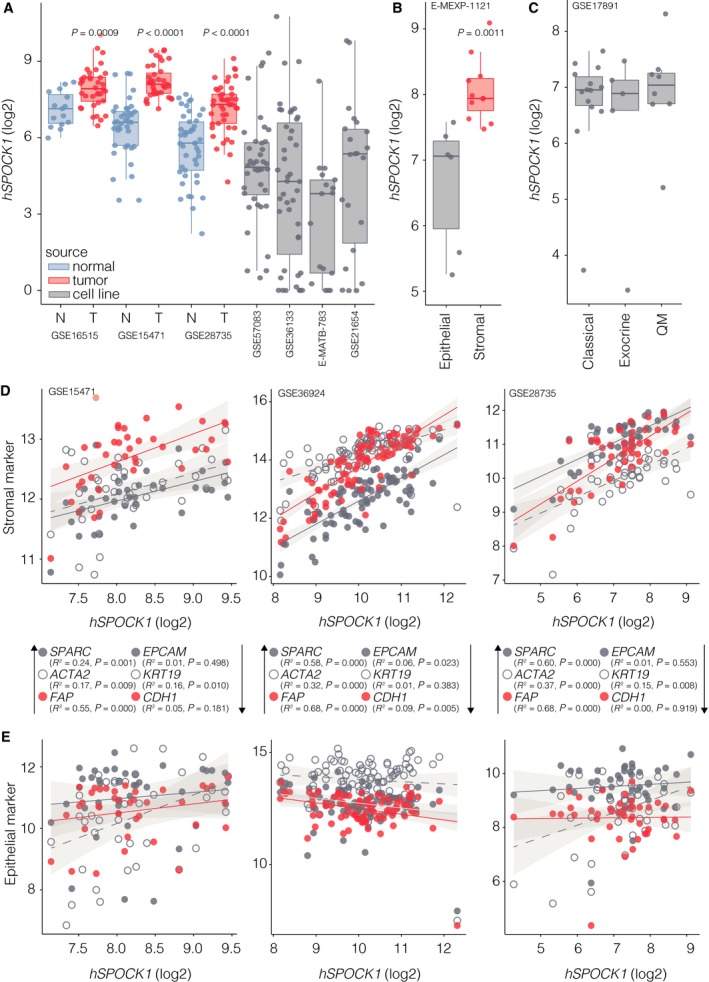
SPOCK1 is upregulated in pancreatic cancer and its expression is confined to the stroma. (A) Indicated microarray gene expression datasets were queried for *SPOCK1* expression levels (Badea *et al*., 2008; Pei *et al*., 2009; Zhang *et al*., 2013). Blue box plots indicate normal (nontumor) pancreas samples, and red box plots indicate tumor samples. Gray box plots show expression in purely epithelial pancreatic cancer cell lines (Barretina *et al*., 2012; Garnett *et al*., 2012; Maupin *et al*., 2010). Dots show individual samples; boxes indicate median with first and third quartiles. Indicated *P*‐values were determined by unpaired two‐tailed Student's *t*‐test. Statistical significance for cell lines versus tumor samples; *P* < 0.0001. (B) *SPOCK1* levels in microdissected epithelial and surrounding tissue are shown (Pilarsky *et al*., 2008). (C) As for panels A–B, on tumor samples classified using the PDAssigner classifier (Collisson *et al*., 2011). Subtypes are indicated on *x*‐axis. (D) Expression levels of stromal activation markers (log2) were correlated with *SPOCK1* expression (on *x*‐axis) (Badea *et al*., 2008; Perez‐Mancera *et al*., 2012; Zhang *et al*., 2013). Solid line indicates linear regression fit line, and shade area indicates standard error confidence bounds. R‐squared (*R* ^2^) linear regression coefficients, determined using the R linear model function, and statistical significance of regression are plotted next to dot color legends. (E) As for panel D, using epithelial marker genes.

Our previous analyses did not exclude the possibility that *SPOCK1* is also expressed in the tumor cells in PDAC. To further delineate the source of *SPOCK1* in human tumors and confirm its expression to be confined to tumor stroma, we performed extensive analysis on publically available gene expression data. First, we assessed *SPOCK1* expression across microarray datasets that include normal pancreas samples and pancreatic cancer tissue (Badea *et al*., 2008; Pei *et al*., 2009; Zhang *et al*., 2013). *SPOCK1* was significantly upregulated in tumor tissue compared to nontumor samples (Fig. 1A). However, when including expression data from purely epithelial PDAC cell lines (Barretina *et al*., 2012; Garnett *et al*., 2012; Maupin *et al*., 2010), *SPOCK1* expression was found significantly lower or absent in these samples, suggesting a stromal expression pattern for *SPOCK1* (Fig. 1A; gray boxplots). These findings were corroborated in microdissected tumor tissue (Pilarsky *et al*., 2008), where *SPOCK1* expression was predominantly expressed in the nonepithelial fraction (Fig. 1B).

Gene expression‐based subgroups have been identified in PDAC. For instance, Collisson *et al*. (2011) demonstrated the existence of three PDAC subtypes, including a poor‐prognosis quasi‐mesenchymal (QM) subtype characterized by EMT‐associated genes. This subgroup identification was predominantly established using tumor cells and microdissected tumor tissue, and the QM signature in the classified samples was therefore not confounded by stromal infiltration. We did not find *SPOCK1* to be significantly higher in the samples classified as QM compared to the other subtypes, establishing that *SPOCK1* expression is stromal rather than a hallmark of tumor cells of a mesenchymal phenotype (Fig. 1C).

In bulk tumor‐derived expression data, we found a very strong correlation of *SPOCK1* expression with markers of activated stroma (Fig. 1D): secreted protein acidic and cysteine rich (*SPARC*), α‐smooth muscle actin (αSMA/*ACTA2*), and fibroblast activation protein. We found no obvious or consistent inverse correlation with tumor cell content as inferred from cytokeratin 19 (KRT19), epithelial cell adhesion molecule (EPCAM) and E‐cadherin (CDH1) expression (Fig. 1E), suggesting that the expression of stromal *activation* markers including *SPOCK1* is not the consequence of increased stromal *content*.

### 3.2 SPOCK1 is a stromal target of TGF‐β in PDAC

**Figure 2:**
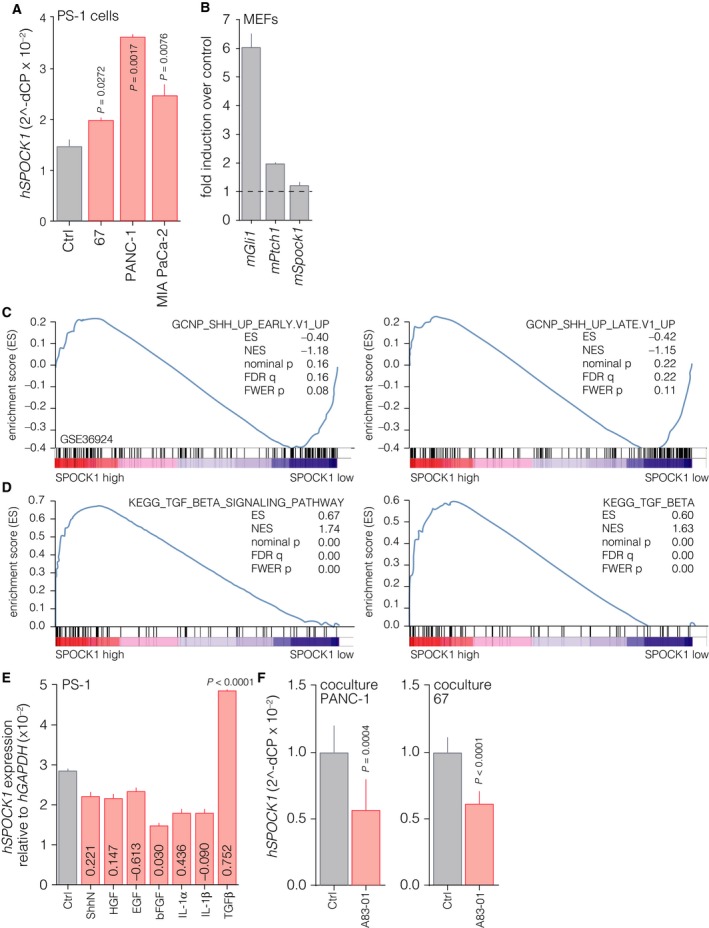
SPOCK1 is a stromal target of tumor cell‐derived TGF‐β in PDAC. (A) PS‐1‐immortalized human stellate cells were cocultured with indicated Venus fluorophore‐expressing PDAC cell lines for 96 h. Cells were subsequently FACS‐sorted and processed for qRT‐PCR for *SPOCK1* and *GAPDH*. Shown is mean ± SEM of *SPOCK1* values relative to housekeeping gene. *P*‐values were determined by unpaired two‐tailed Student's *t*‐test comparing control to tumor cell cocultures. (B) MEFs were serum‐starved in 0.5% FBS and treated with ShhN for 48 h. Transcript analysis for genes indicated on *x*‐axis was performed. Bars show mean induction relative to control (set to 1) ± SEM, *n* = 3. (C) GSEA was performed using indicated gene expression dataset (Perez‐Mancera *et al*., 2012). Samples were dichotomized by median *SPOCK1* expression. Gene sets for SHH signaling were from Zhao *et al*. (2002). See also Materials and methods, Section 2.2. (D) GSEA was performed as for C, using two KEGG‐derived TGF‐β‐related gene sets. (E) PS‐1 cells were starved with 0.5% FBS for 24 h and subsequently treated with the indicated ligands for 48 h. ShhN, 1 : 4 diluted 293T supernatant; HGF, 10 ng·mL^−1^; EGF, 50 ng·mL^−1^; bFGF, 10 ng·mL^−1^; IL‐1α, 10 ng·mL^−1^; IL‐1β, 10 ng·mL^−1^; TGF‐β, 5 ng·mL^−1^. Number in bars indicates *r*‐value of correlation of *SPOCK1* with transcripts coding for ligands used (dataset GSE28735 (Zhang *et al*., 2013)). Bars show mean *SPOCK1* levels relative to *GAPDH* ± SEM, *n* = 3. Indicated *P*‐value was determined by unpaired two‐tailed Student's *t*‐test comparing control and TGF‐β condition only. (F) PS‐1 cells were cocultured with indicated cancer cells and serum‐starved as for E. TGF‐β pathway inhibitor A38‐01 was used at 1 μm. Bars show mean *SPOCK1* levels relative to *GAPDH* ± SEM. At least triplicates are shown. Indicated *P*‐values were determined by unpaired two‐tailed Student's *t*‐test comparing control and A83‐01.

To experimentally confirm that indeed SPOCK1 is expressed in tumor‐instructed stromal cells, we cocultured an immortalized human pancreatic stellate cell line (PS‐1; Froeling *et al*., 2009) with a previously established PDAC cell lines (PANC‐1 and MIA PaCa‐2) as well as a primary patient‐derived tumor cell line established in our laboratory (67; Damhofer *et al*., 2015). Prior to the experiment, tumor cells were transduced with a fluorophore to allow FACS‐based sorting and qRT‐PCR on the stellate cells following the coculture (Fig. 2A). In all cocultures, an induction of SPOCK1 expression in stellate cells was observed, confirming it to be a consistent target of tumor cell‐derived signals in stromal cells.

To further characterize the association of SPOCK1 expression with tumor biological processes specific to PDAC, extensive additional GSEA on several gene sets was performed using previously identified tissue‐ or cell type‐specific gene signatures (Table S1A) (Moffitt *et al*., 2015). Of these, *activated stroma* genes showed best enrichment across several datasets dichotomized for *SPOCK1* expression. This was corroborated using previously established gene sets for PDAC stromal infiltration and extracellular matrix in these same analyses (Table S1B). The screen in which SPOCK1 was identified relied on a blocking strategy testing the requirement for SHH ligand, but left its sufficiency untested (Damhofer *et al*., 2013). When MEFs (as also used for (Damhofer *et al*., 2013)) were treated with SHH ligand, induction of the target genes *Gli1* and *Ptch1* was observed but no *Spock1* induction was detected (Fig. 2B). Gene set enrichment analysis (GSEA) using previously established gene sets for SHH signaling did not show convincing positive enrichment scores in tumors with high *SPOCK1* expression (Fig. 2C) (Zhao *et al*., 2002). In contrast, GSEA with two Kyoto Encyclopedia of Genes and Genomes (KEGG) gene sets for TGF‐β, a ligand known to mediate tumor–stroma crosstalk across many cancer types, yielded good enrichment scores. This suggested this ligand to be a likely candidate inducer of *SPOCK1* in PDAC stroma (Fig. 2D).

To functionally establish the relative potency of TGF‐β to induce SPOCK1 compared to other ligands known to mediate tumor–stroma crosstalk in PDAC, we applied a panel of such ligands to the PS‐1 cells and determined *SPOCK1* levels by qRT‐PCR (Fig. 2E). For each ligand, we also determined their correlation with *SPOCK1* on microarray expression data (*R* ^2^ values plotted in the bars). Surprisingly, of all ligands tested, only TGF‐β was able to induce *SPOCK1* expression. Inhibitor experiments on cocultures of PANC‐1 or 67 primary cells with stellate cells confirmed the role of TGF‐β in driving SPOCK1 expression; inhibition of the TGF‐β pathway using the small‐molecule inhibitor A83‐01 efficiently blocked SPOCK1 expression (Fig. 2F). These data suggest that the identification of *SPOCK1* in our initial *in vitro* screen relied on a combination of ligands in which SHH ligand was required but not sufficient, and that TGF‐β ligand is likely sufficient to drive robust *SPOCK1* expression.

### 3.3 Stromal SPOCK1 affects collagen deposition

**Figure 3:**
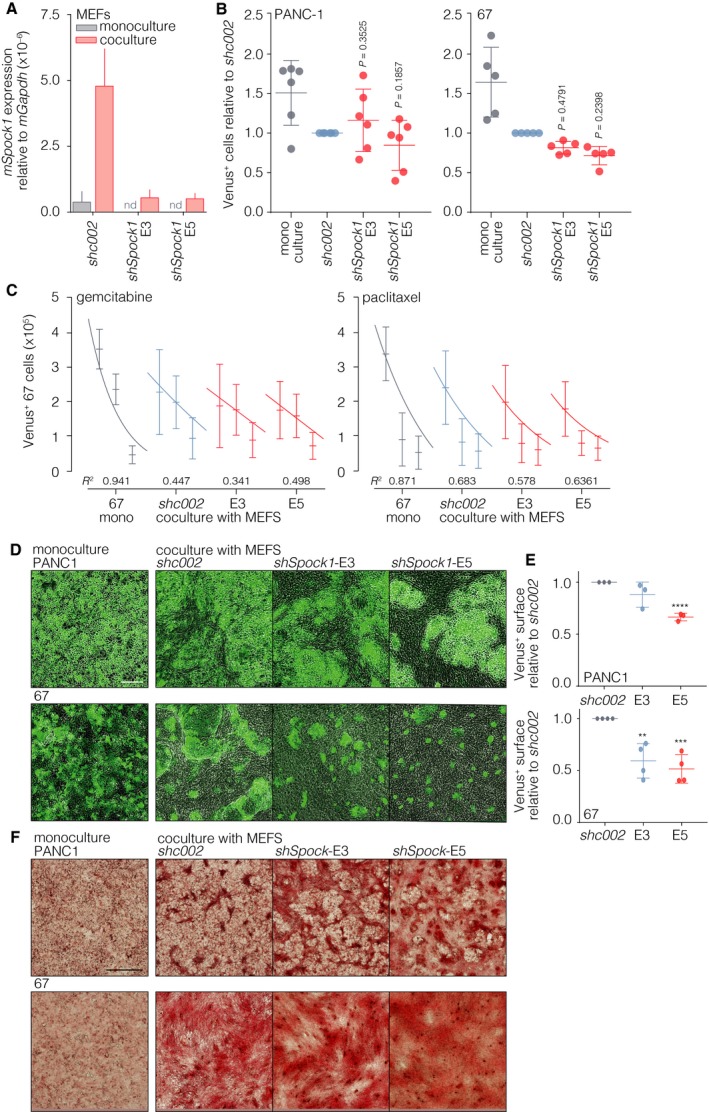
Stromal SPOCK1 affects collagen deposition. (A) MEFs were transduced with a control scrambled shRNA (*shc002*) construct or with constructs with shRNA sequences against *Spock1*. All five TRC clones showed efficient knockdown (average efficiency 88.3 ± 8.5% SEM, *P* = 0.01 by one‐way ANOVA). Two clones were chosen for further experimentation: E3 and E5. Cells were cocultured with PANC‐1 cells to induce *Spock1* to detectable levels. After 5‐day coculture, cells were harvested and qRT‐PCR was performed using species‐specific primers. Bars show mean *Spock1* levels relative to *Gapdh* ± SEM, *n* = 3. Difference between groups; *P* = 0.040. (B) MEFs were cocultured with PANC‐1 or 67 PDAC cell lines for 7 days and harvested for counting by bead‐normalized FACS (see also Materials and methods, Section 3.4). Shown are values normalized to *shc002* control (set to 1), means ± SEM, *n* = 5 or 6. Indicated *P*‐value was determined by unpaired two‐tailed Student's *t*‐test comparing *shc002* control and knockdown conditions. (C) Cultures with 67 primary PDAC cells grown in total for 7 days as for B were treated with 0, 2, and 10 nm gemcitabine or 0, 2, and 2.5 nm paclitaxel for 5 days and the number of tumor cells was counted by FACS. Shown are means ± SEM, *n* = 2. Curves were fitted using GraphPad Prism; *R* ^2^ indicates goodness of Fit. (D) Cocultures as for B were imaged by brightfield and fluorescence microscopy. Overlays of both channels are shown. Scalebar: 200 μm. (E) Quantification of Venus‐positive area relative to *shc002* control. Statistical significance was determined by unpaired two‐tailed Student's *t*‐test comparing to *shc002*. **P < 0.01; ***P < 0.005; ****P < 0.0001, *n* = 3. (F) As for B followed by picrosirius red staining and visualization by brightfield microscopy. Scalebar: 200 μm.

The identification of SPOCK1 as a stromal target for tumor cell‐derived ligands and its prognostic power raise the question whether it has a functional role in driving tumor biology. We applied lentiviral shRNA silencing of *Spock1* (*shSpock1)* in MEFs and verified knockdown by qRT‐PCR using species‐specific primers (Fig. 3A). *Spock1* levels were almost undetectable in monoculture of these MEFs, and coculturing MEFs with tumor cells was necessary to induce expression. By doing so, we were able to show efficient knockdown of *Spock1* by hairpin clones E3 and E5.

To test the effect of stromal *Spock1* ablation on tumor growth, Venus‐expressing tumor cells and *shSpock1* MEFs were cocultured in two‐dimensional culture and the number of tumor cells was counted by bead‐normalized FACS (Fig. 3B). There was no effect of *shSpock1* on the number of PANC‐1 tumor cells in cocultures, and *shSpock1* cocultures with 67 primary cells showed only a modest decrease in the number of tumor cells. Next, we tested the effect of coculturing on resistance against therapeutics commonly used against PDAC, gemcitabine and paclitaxel (Burris *et al*., 1997; Goldstein *et al*., 2015; Von Hoff *et al*., 2013). PANC‐1 cells were resistant to the concentrations used, and higher concentrations could not be used as these were toxic to the MEFs (Fig. S2). However, as expected, cocultures of 67 primary PDAC cells with control‐silenced MEFs blunted the effect of chemotherapeutics as compared to 67 primary cell monocultures (Fig. 3C). However, no effect of *shSpock1* was observed in either coculture, and we concluded that chemoresistance is not the mechanism through which stromal SPOCK1 expression contributes to poor prognosis in patients.

Prior to harvesting for FACS, cocultures were imaged, and from these images, a much smaller size of tumor cell colonies grown together with the *shSpock1* MEFs was immediately apparent (cf. Fig. 3D). These smaller colonies were seemingly incongruent with the unchanged number of tumor cells as counted by FACS (Fig. 3B), and implied that more tumor cells occupied a smaller surface area in these *Spock1‐*knockdown cocultures. This observation, and the known interactions of glycoproteins like SPOCK1 with the ECM, led us to hypothesize that SPOCK1 could act on the extracellular matrix and thereby indirectly affect the growth pattern of the tumor cells. When collagen, a major constituent of the PDAC extracellular matrix, was visualized in the cocultures by picrosirius red, marked differences were indeed apparent (Fig. 3F). In *shSpock1* cocultures, collagen fiber patterns were more diffuse than in the control cocultures. Furthermore, the MEF monolayer often grew on top of the tumor cell colonies in the *shSpock1* cocultures (as can be seen from the foci of collagen in Fig. 3E, see also the distribution of tumor cell colonies in Fig. 3D), whereas in control cocultures, the tumor colonies typically remained uncovered. These data suggest that by acting on the extracellular collagen, stromal SPOCK1 affects tumor cell growth and dispersal.

### 3.4 SPOCK1 affects invasive tumor cell growth

**Figure 4:**
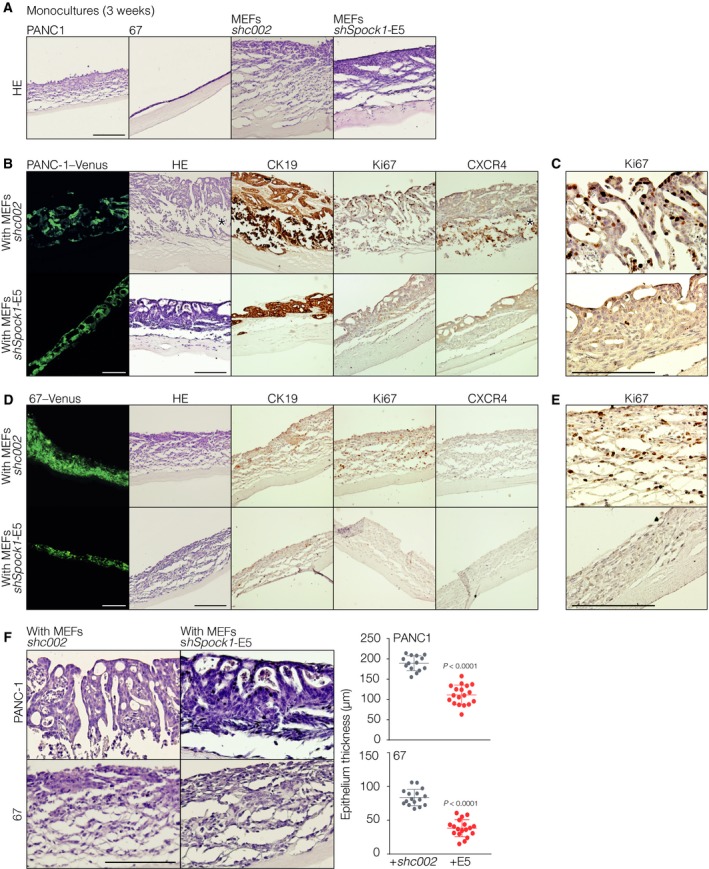
SPOCK1 affects invasive tumor cell growth in organotypic cocultures. (A) Organotypic monocultures for the indicated cell lines were grown for 3 weeks and processed for routine histopathology by hematoxylin and eosin (HE). Scalebar: 200 μm. (B) Organotypic (co)cultures of PANC‐1 cells with indicated cells were grown for 3 weeks and cultures were processed for immunofluorescence for transduced fluorophore (Venus), HE, and immunohistochemistry for indicated proteins. Percentages in Ki67 represent nuclei positive, *n* = 2. Top row is using *shc002* control MEFs; bottom row is with *shSpock1* MEFs. Scalebar: 200 μm. (C) Higher magnification of Ki67‐stained specimens. (D,E) As for B, using 67 primary PDAC cells. (F) Higher magnification of HE‐stained PANC‐1 and 67 cocultures, with quantification. *P*‐values were determined by unpaired two‐tailed Student's *t*‐test comparing *shc002* control and *E5*‐knockdown clone.

To more conclusively address the impact of stromal SPOCK1 on tumor cell growth patterns, and for lack of a feasible *in vivo* model to study this, we turned to advanced organotypic culturing models (Froeling *et al*., 2010). These cultures rely on an air–liquid interface, optimized extracellular matrix composition, and nutrient gradients to model conditions in tissue. In addition, this method allows cocultures to grow over much longer periods of time than two‐dimensional equivalents. In organotypic monocultures grown for 3 weeks, we observed differences in the growth patterns of the tumor cell lines as expected (Fig. 4A); PANC‐1 cells grew as a thick layer of epithelium, whereas the 67 primary cells covered the collagen matrix with a single layer of cells. Interestingly, the *shSpock1* MEF monocultures grew much less invasively than the control MEFs did, suggesting that these cells interact with matrix differently. The effects of MEF monocultures on the collagen matrix were not observed in short‐term organotypic cultures (1 week; Fig. 3A).

In PANC‐1/MEF organotypic cocultures, a fairly well‐differentiated layer of epithelium could be observed (Fig. 4B). The thickness of this layer was much reduced in cocultures with *shSpock1* MEFs, suggesting a role for stromal SPOCK1 in facilitating tumor growth. This was further supported by a reduced number of Venus‐ and CK19‐positive cells. Proliferation, measured by IHC for Ki67, was reduced in the *shSpock1* MEF cocultures relative to control‐silenced cocultures (Fig. 4B,C, 15.89 ± 1.13% versus 37.4 ± 3.20%, *P* < 0.0001 by Student's *t*‐test). The 67 primary cell cocultures showed less tumor cell growth in general, but the effect of *shSpock1* in these cultures mirrored that seen with the PANC‐1 cells. A strong reduction in proliferative index was observed in the absence of stromal SPOCK1 (Fig. 4C–E, 14.83 ± 1.21% versus 38.25 ± 1.26%, *P* < 0.0001).

To reveal the effects of *shSpock1* on collagen composition in these organotypic cocultures, we used picrosirius red staining and polarized light microscopy, which allows the identification of the types of collagen types (Lattouf *et al*., 2014). This analysis revealed that indeed, control and *shSpock1* cocultures exerted differential effects on the collagen matrix (Fig. S3B), suggesting that Spock1 could alleviate the constraints exerted on tumor cells by acting on the extracellular matrix. Indeed, in the control PANC‐1 organotypic cocultures, we observed a tumor cell population that had grown deep into the collagen matrix (Fig. 4B, indicated by asterisks as well as by the perturbed structure following sectioning). The increased expression of CXCR4 in this population suggests that these cells constitute a relatively invasive population of tumor cells. In the *shSpock1* cocultures, this population was notably absent, which implies that stromal SPOCK1 also enables invasive growth of tumor cells. The organotypic coculture data together with the analyses of collagen composition show that SPOCK1 in stromal cells functions to modify the collagen matrix and thereby facilitates the invasive growth of tumor cells. In the absence of SPOCK1, tumor cells are more mechanically restricted by the matrix, possibly explaining the prognostic value of SPOCK1 in PDAC.

## 4 Discussion

The contributions of the stroma to PDAC are strongly diverse and now well recognized to include opposing effects (systematically reviewed in Bijlsma and van Laarhoven (2015)). A large number of studies have demonstrated tumor‐promoting contributions of the stroma to PDAC growth, chemoresistance, and metastasis. These effects are mediated, for instance, through the mechanical restrictions that the dense stroma exerts on tumor perfusion, or by the mixture of extracellular stromal signals that shape a complex niche for tumor cells to exist in. However, it has now become clear that the stroma also holds tumor‐restraining properties. This was most apparent from recent unsuccessful clinical trials using stroma‐targeting drugs in PDAC (BusinessWire, 2014; Kim *et al*., 2014). Experimental work demonstrated that the ablation of stroma from established tumors strongly increased aggressive tumor growth (Lee *et al*., 2014; Ozdemir *et al*., 2014; Rhim *et al*., 2014). It will therefore become important to identify and target specific stromal genes that act tumor‐promoting, while leaving the tumor‐restricting features of the stroma intact.

In this study, we have addressed tumor‐promoting stromal factors and identified SPOCK1 as a mediator of extracellular matrix remodeling and invasive tumor growth. We demonstrated that stromal SPOCK1 does not directly affect the chemoresistance of tumor cells, but that stromal SPOCK1 does strongly contribute to tumor growth and invasiveness in three‐dimensional cultures. Interestingly, these tumor‐promoting effects could not be revealed in classical two‐dimensional cocultures. It is possible that the longer culturing made possible by organotypic cocultures is responsible for this, but it is also possible that the tumor–stroma interaction needs a three‐dimensional configuration for the growth‐promoting effects of SPOCK1 to become apparent.

We have shown that SPOCK1 affects tumor cells by acting on the extracellular collagen, thereby facilitating tumor cell expansion. This finding fits well with the notion that stiffening of the extracellular matrix acts on the proliferation, migration, and adhesion of tumor cells (Butcher *et al*., 2009). Although signaling mediated by collagen I – the major component of extracellular matrix – has been demonstrated to increase the clonogenic capacity of pancreatic tumor cells under treatment with 5‐FU, allowing chemoresistant clones to grow out (Armstrong *et al*., 2004), we did not observe an enhancement of the efficacy of gemcitabine and paclitaxel following the ablation of SPOCK1. This implies that the effects of *shSpock1* in our experiments are mediated through mechanical properties rather than matrix‐derived signaling molecules.

An important question left unexplored in this study is the correlation of stromal SPOCK1 expression with important tumor‐promoting stromal features in patient tumor material. Attempts by us to assess stromal SPOCK1 by IHC revealed staining patterns that were incongruent with the molecular data as shown in this manuscript, and we propose that future studies on large patient cohorts using novel, more specific, antibodies or methods like *in situ* hybridization should be used to address this.

The SPARC protein family, of which SPOCK1 is a member, has diverse functions but all appear to be involved in the regulation of extracellular matrix aggregation and degradation. The role of SPARC in tumorigenesis and growth varies. In PDAC, SPARC signaling appears to be associated with tumor growth suppression *in vitro* (Sato *et al*., 2003). Clinical studies, however, showed a correlation between high stromal SPARC expression and poor prognosis (Gundewar *et al*., 2015). The results from our study (i.e., that depletion of stromal SPOCK1 inhibits tumor cell proliferation and invasion) are in apparent contrast to what is found for stromal SPARC expression in PDAC, emphasizing the complexity of the function of these protein family members, and the PDAC extracellular matrix in general.

Previous publications have demonstrated a role for SPOCK1 in cancer types such as breast cancer (Fan *et al*., 2016), prostate cancer (Chen *et al*., 2016; Yang *et al*., 2015), glioblastomas (Yu *et al*., 2016), urothelial carcinomas (Ma *et al*., 2016), ovarian cancer (Zhang *et al*., 2015), esophageal squamous cell carcinomas (Song *et al*., 2015), gallbladder cancer (Shu *et al*., 2015), lung cancer (Miao *et al*., 2013), and hepatocellular carcinomas (Li *et al*., 2013). All these studies focused on the epithelial fraction. Instead, we find that in PDAC, SPOCK1 is confined to the stromal compartment but indirectly affects the proliferation and invasion of tumor cells. This indirect, extracellular matrix‐mediated effect of SPOCK1 could also explain its correlation with poor prognosis in other (non‐PDAC) tumor types.

## 5 Conclusion

In conclusion, we have identified SPOCK1 as a stromal protein that mediates tumor‐promoting effects by acting on the extracellular matrix, and propose that it serves as consistent mediator of tumor‐derived TGF‐β signaling across all cases of PDAC despite the heterogeneous genetic makeup of the tumor compartment. The identification of specific tumor‐promoting stromal proteins can aid in the development of novel treatment combinations, most likely on a backbone of cytotoxic drugs. Furthermore, the expression of such proteins can identify patients that harbor stroma of a particularly malignant activation status, and be used for stratification.

## Author contributions

VLV and MFB conceived and designed the project. VLV, HD, CW, AS, and HMK acquired the data. VLV, JPM, HWL, and MFB analyzed and interpreted the data. VLV and MFB wrote the manuscript.

## Conflict of interests

The authors declare no conflict of interest. MFB has received research funding from Celgene. HWL has acted as a consultant for Eli Lilly and Company, and Nordic Pharma Group, and has received research grants from Amgen, Bayer Schering Pharma AG, Celgene, Eli Lilly and Company, GlaxoSmithKline Pharmaceuticals, Nordic Pharma Group, and Roche Pharmaceuticals. None of these authors were involved in drafting of the manuscript.

## Supporting information


**Fig. S1.** Association of *SPOCK1* expression with survival in publicly available expression datasets.
**Fig. S2.** Treatment of two‐dimensional cocultures of PANC‐1 cells.
**Fig. S3.** Organotypic monocultures.
**Table S1.** Gene set enrichment analyses for SPOCK1‐associated signatures.
