# Supplementary material for: Stromal SPOCK1 supports invasive pancreatic cancer growth
Source: Mol Oncol. 2017 Jun 5;11(8):1050–64. doi: 10.1002/1878-0261.12073 (PMC5537700; doi:10.1002/1878-0261.12073)
Supplement: Supplementary file 1 — Fig. S1. Association of SPOCK1 expression with survival in publicly available expression datasets. Fig. S2. Treatment of two‐dimensional cocultures of PANC‐1 cells. Fig. S3. Organotypic monocultures. Table S1. Gene set enrichment analyses for SPOCK1‐associated signatures. [file MOL2-11-1050-s001.pdf]

Supplementary material for

Stromal SPOCK1 impacts on invasive pancreatic cancer growth

Veronique L Veenstra[a], Helene Damhofer[a,b], Cynthia Waasdorp[a], Anne Steins[a], Hemant M Kocher[c], Jan Paul Medema[a], Hanneke W van Laarhoven[d], and Maarten F Bijlsma[a,*]

[a]Laboratory for Experimental Oncology and Radiobiology, Center for Experimental and Molecular Medicine, Academic Medical Center and Cancer Center Amsterdam, Meibergdreef 9, 1105 AZ Amsterdam, The Netherlands

[b] Currently at the Biotech Research & Innovation Centre, Copenhagen, Denmark

[c] Centre for Tumour Biology, Barts Cancer Institute, Queen Mary University of London, UK.

[d]Department of Medical Oncology, Academic Medical Center, University of Amsterdam, Amsterdam, the Netherlands

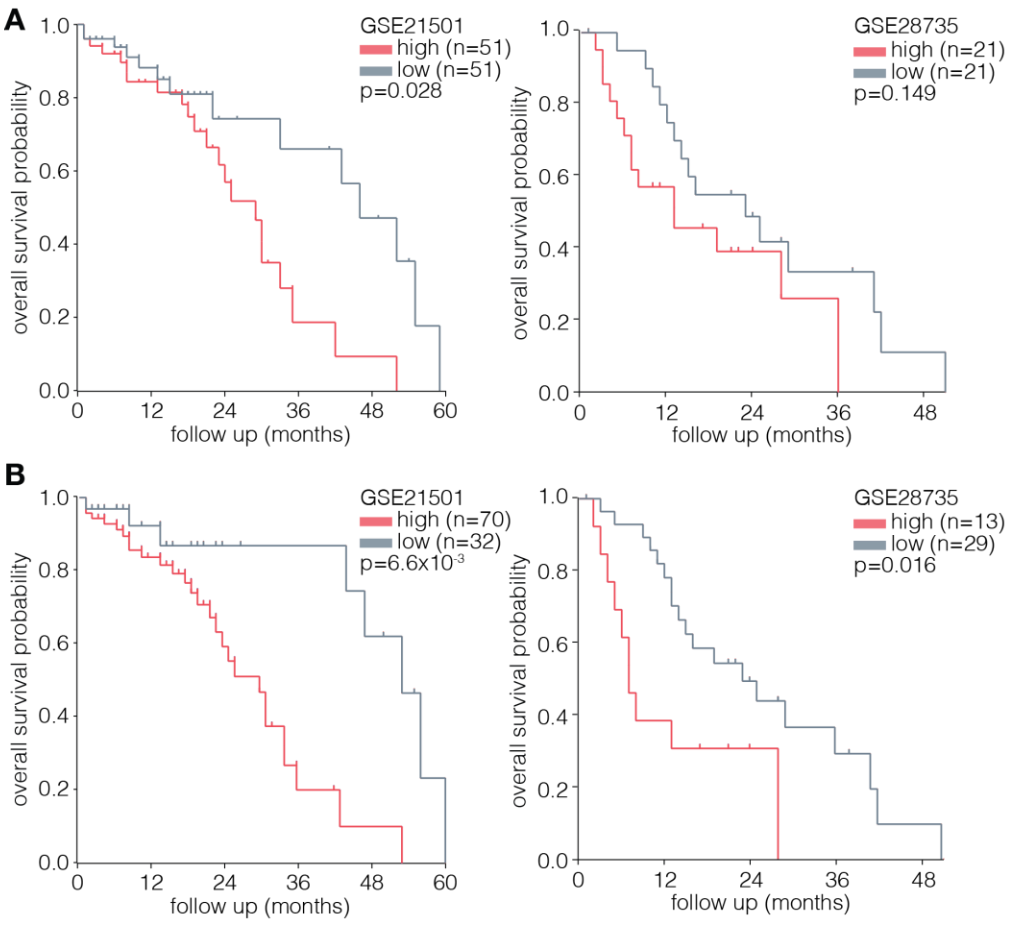

Supplementary Figure 1 – Association of *SPOCK1* expression with survival in publicly available expression datasets. **A.** Patients in the indicated gene expression datasets (1,2) were dichotomized by median *SPOCK1* expression and overall survival was used for Kaplan Meier analysis. Indicated p value is log-rank tested. **B.** As for A, dichotomization by the *SPOCK1* expression that yields best separation on survival (scan). Number of patients in each group is indicated. Indicated p value is log-rank tested.

	GSE15471		GSE36924		GSE28735		GSE 16515	
	NES	FWER p	NES	FWER p	NES	FWER p	NES	FWER p
F1_Liver	-1,14	0,59	-1,36	0,37	-1,29	0,74	-0,96	0,98
F2	-0,99	0,76	1,13	0,87	-0,75	1,00	-1,21	0,81
F3_Exocrine	-0,96	0,60	-1,25	0,68	-1,33	0,67	-1,38	0,54
F4_CellCycle	1,22	0,85	-1,41	0,23	1,59	0,10	1,78	**0,02**
F5_ActivatedStroma	1,80	**0,01**	1,48	0,12	1,47	0,30	1,60	0,14
F6_BasalLike	1,51	0,19	-0,72	1,00	1,38	0,53	0,88	0,99
F7_Immune	-1,26	0,78	-0,64	1,00	-0,98	0,99	-0,68	1,00
F8_Classical	0,71	1,00	-1,40	0,24	0,58	1,00	-1,18	0,85
F9_Lung	-0,98	0,66	1,36	0,39	0,80	1,00	0,92	0,98
F10	0,73	1,00	1,07	0,92	1,48	0,28	-1,20	0,82
F11_Muscle	-1,36	0,54	1,52	0,08	-1,11	0,95	-1,25	0,75
F12_Endocrine	-0,94	0,56	-1,19	0,77	-1,36	0,59	-1,41	0,47
F13_NormalStroma	-1,17	0,88	1,35	0,40	-1,00	0,98	-0,52	1,00
F14	-1,15	0,90	-1,13	0,87	0,70	1,00	-1,21	0,81
stroma_simeone	1,24	0,56	1,53	**0,01**	1,32	0,39	1,44	0,27
stromal_yoshihara	0,67	0,97	1,55	**0,00**	0,95	0,76	1,14	0,63
extracellular_matrix_part	0,96	0,84	1,63	**0,00**	1,54	0,10	0,93	0,82
collisson_classical_geneset	0,78	0,94	-1,38	0,15	-0,57	0,93	-1,22	0,55
immune_infiltration	0,94	0,85	1,09	0,67	-0,80	0,85	0,60	0,96
gcnp_shh_up_early.V1_up	1,11	0,72	-1,18	0,53	1,45	0,20	1,49	0,22
gcnp_shh_up_late.V1_up	1,07	0,75	-1,15	0,59	1,19	0,55	1,55	0,15
kegg_tgfb	0,00	0,92	1,63	**0,00**	1,11	0,63	0,93	0,81
kegg_tgf_beta_signaling_pathway	0,80	0,83	1,73	**0,00**	1,30	0,42	1,06	0,71

Supplementary Table S1 – Gene set enrichment analyses for SPOCK1-associated signatures.
A. Samples from indicated sets (2-5) were dichotomized by median *SPOCK1* expression and GSEA with factors from (6) were tested. Indicated are normalized enrichment scores (NES) and family-wise error rate corrected significance tests (FWER p). Color of cells indicates high NES (blue to red) or low p-value. **B,** As for A, using gene sets selected from publications, the Molecular Signature Database (MSigDB), and the Kyoto Encyclopedia of Genes and Genomes (KEGG) gene sets.

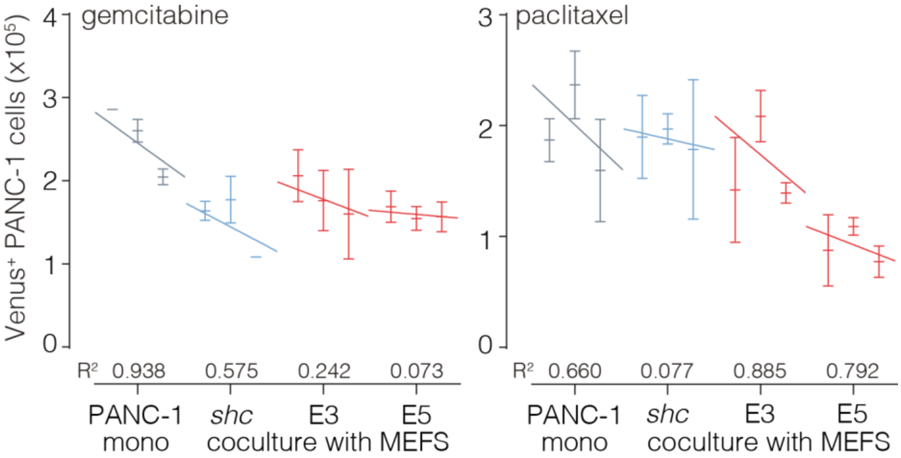

Supplementary Figure 2 – Treatment of two-dimensional cocultures of PANC-1 cells.

PANC-1 cells were cocultured with MEFs for 7d and harvested for counting by bead-normalized FACS (see also Materials and methods section 2.9 and main Fig. 3c). Cells were treated with 0, 2, and 10 nM gemcitabine or 0, 2, and 2.5 nM paclitaxel and number of tumor cells was counted by FACS. Shown are means ±S.E.M., n=2. Curves were fitted using Graphpad Prism.

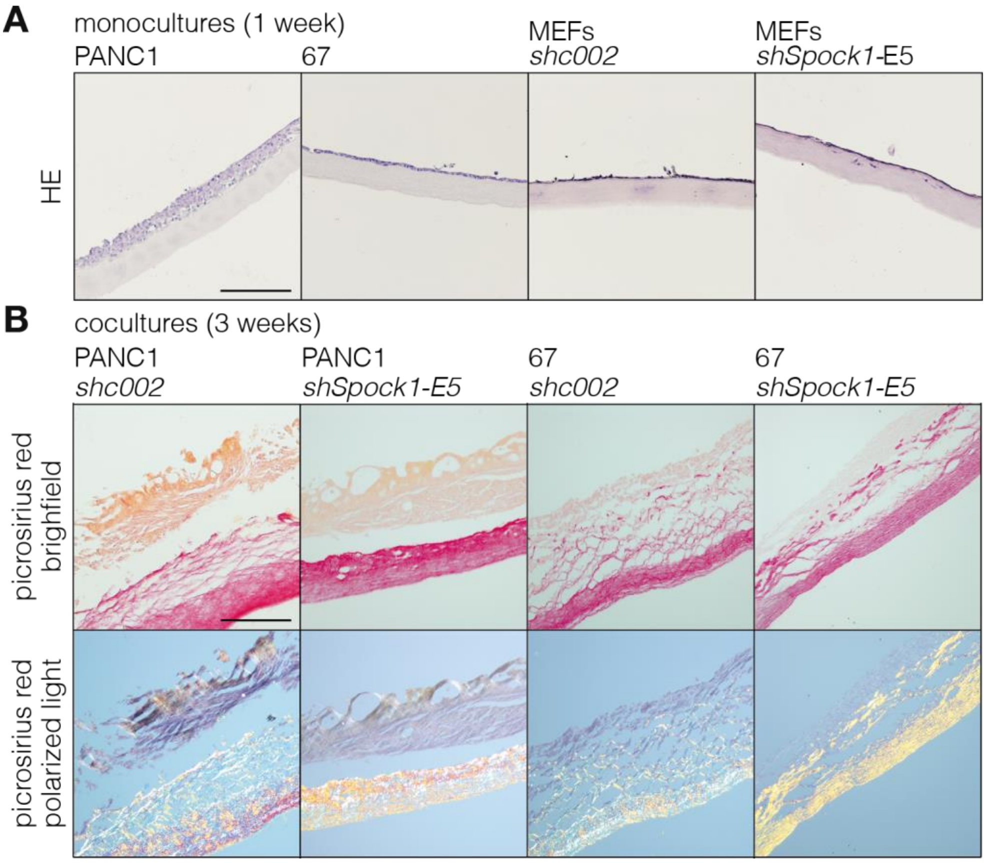

Supplementary Figure 3 – Organotypic monocultures.

A. The indicated cell lines were grown for 1 week (as opposed to 3 weeks for Fig. 4) and processed for routing histopathology by hematoxylin and eosin (HE). Scalebar; 200 µm.

B. Organotypic cocultures were grown for 3 weeks and Picrosirius red staining was performed. Microscopy was performed using brightfield (top row) or polarized lighting (bottom row).

REFERENCES

1. Stratford JK, Bentrem DJ, Anderson JM, Fan C, Volmar KA, Marron JS*, et al.* A six-gene signature predicts survival of patients with localized pancreatic ductal adenocarcinoma. PLoS medicine **2010**;7(7):e1000307.
2. Zhang G, He P, Tan H, Budhu A, Gaedcke J, Ghadimi BM*, et al.* Integration of metabolomics and transcriptomics revealed a fatty acid network exerting growth inhibitory effects in human pancreatic cancer. Clin Cancer Res **2013**;19(18):4983-93.
3. Badea L, Herlea V, Dima SO, Dumitrascu T, Popescu I. Combined gene expression analysis of whole-tissue and microdissected pancreatic ductal adenocarcinoma identifies genes specifically overexpressed in tumor epithelia. Hepato-gastroenterology **2008**;55(88):2016-27.
4. Pei H, Li L, Fridley BL, Jenkins GD, Kalari KR, Lingle W*, et al.* FKBP51 affects cancer cell response to chemotherapy by negatively regulating Akt. Cancer cell **2009**;16(3):259-66.
5. Perez-Mancera PA, Rust AG, van der Weyden L, Kristiansen G, Li A, Sarver AL*, et al.* The deubiquitinase USP9X suppresses pancreatic ductal adenocarcinoma. Nature **2012**;486(7402):266-70.
6. Moffitt RA, Marayati R, Flate EL, Volmar KE, Loeza SG, Hoadley KA*, et al.* Virtual microdissection identifies distinct tumor- and stroma-specific subtypes of pancreatic ductal adenocarcinoma. Nature genetics **2015**;47(10):1168-78.
